# Supplementary material for: Comprehensive Annotation and Functional Exploration of MicroRNAs in Lettuce
Source: Front Plant Sci. 2021 Dec 24;12:781836. doi: 10.3389/fpls.2021.781836 (PMC8739914; doi:10.3389/fpls.2021.781836)
Supplement: Supplementary file 3 [file Data_Sheet_9.ZIP › GSM2230752.plot/Lsa-miRN1651_Lsat_1_v5_gn_2_106441.1_2383_TPlot.pdf]

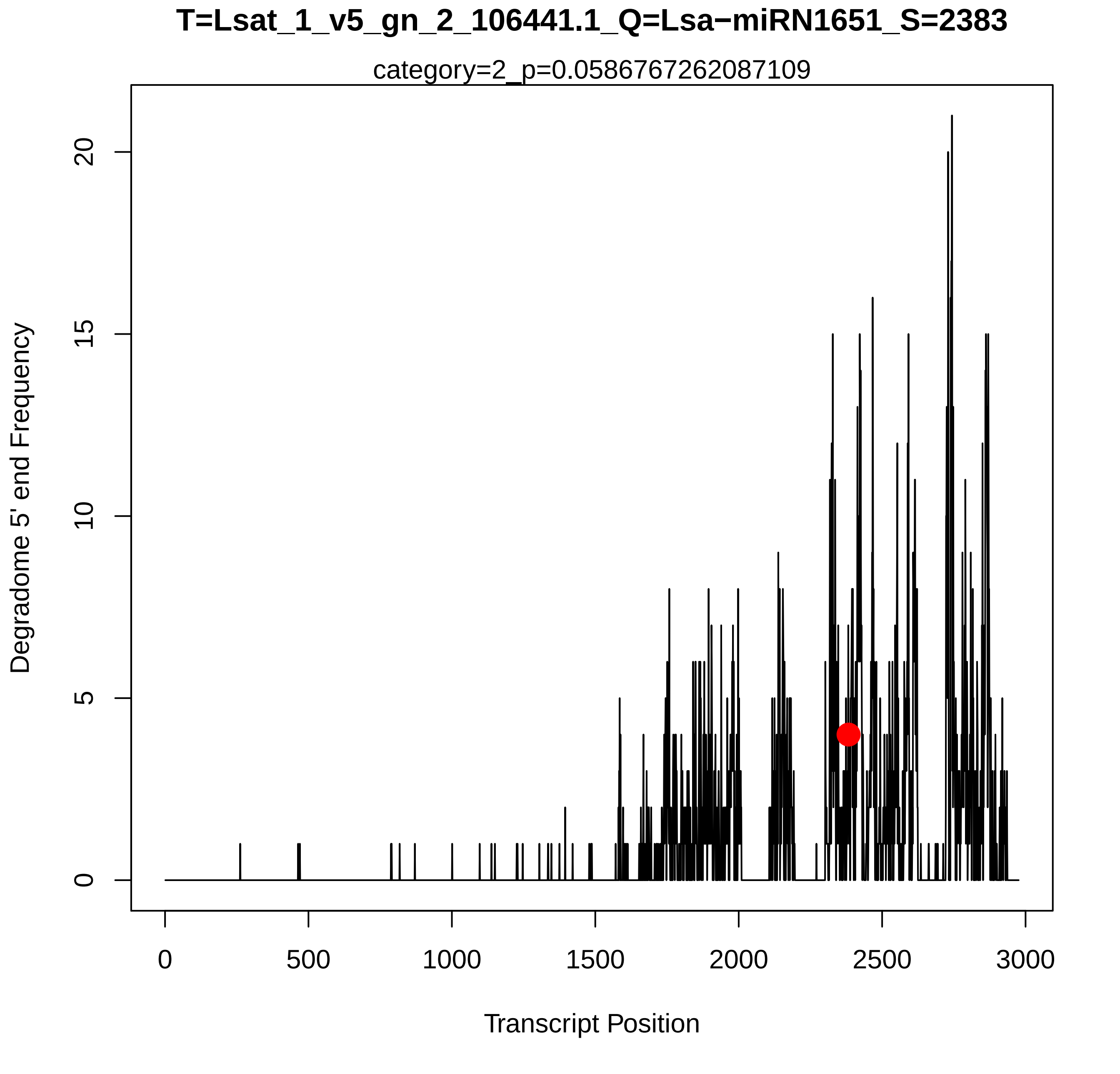
T=Lsat_1_v5_gn_2_106441.1_Q=Lsa−miRN1651_S=2383
category=2_p=0.0586767262087109
Degradome 5' end Frequency
Transcript Position
0
5
10
15
20
0
500
1000
1500
2000
2500
3000
